# Rapid Desensitization for Hypersensitivity Reactions to Chemotherapeutic Drugs; A Case Series

**DOI:** 10.22037/ijpr.1999.1100664

**Published:** 2019

**Authors:** Delara Babaie, Bibi Shahin Shamsian, Nader Momtazmanesh, Hamidreza Godarzipour, Mehrdad Amirmoini, Bahram Bashardoust, Masoomeh Ebrahimi, Mahdieh Vahedi, Reza Ghaemi, Mehrnaz Mesdaghi

**Affiliations:** a *Department of Immunology and Allergy, Mofid Children’s Hospital, Shahid Beheshti University of Medical Sciences, Tehran, Iran.*; b *Pediatric Congenital Hematologic Disorders Research Center, Mofid Children’s Hospital, Shahid Beheshti University of Medical Sciences, Tehran, Iran.*; c *Department of Pediatric Hematology and Oncology, Loghman-e- Hakim Hospital, Shahid Beheshti University of Medical Sciences, Tehran, Iran. *; d *Department of Pediatrics, Loghman-e- Hakim Hospital, Shahid Beheshti University of Medical Sciences, Tehran, Iran. *; e *Department of Pediatrics, Mofid Children’s Hospital, Shahid Beheshti University of Medical Sciences, Tehran, Iran.*; f *Department of Pediatrics, Urmia University of Medical Sciences, Urmia, Iran.*

**Keywords:** Adverse drug reaction, Cancer chemotherapy drugs, Drug hypersensitivity syndromes, Drug desensitization

## Abstract

Usage of cancer chemotherapeutics drugs can be associated with adverse drug reactions. When IgE-mediated drug reactions are formed following administration of a chemotherapeutics drug that is a drug of choice, drug desensitization protocols can be helpful. HSR can be allergic or nonallergic, but the clinical manifestations are similar. RDD is effective when used appropriately, however it is often over utilized instead of performing a drug challenge. RDD is both an acceptable approach and a high-risk treatment modality in patients, in whom the offending agent is the first choice in chemotherapy. The safety of this modality has been acceptable in large studies. The side effects are often less frequent and less severe by repeating the protocol. We present 4 cases of successful desensitization in cancer patients, who have developed IgE- mediated reactions to their major chemotherapy drug.

## Introduction

Adverse drug reactions are frequent and occur in 10 to 20% of hospitalized patients and approximately 7% of the general population (1). In the last decades, the frequency of hypersensitivity reactions (HSR) has considerably increased ,especially in patients with cancer and chronic inflammatory diseases (2). HSRs are a spectrum of clinical manifestations from mild cutaneous reactions such as flushing, pruritus, and urticaria, as well as angioedema to more severe reactions including cardiovascular manifestations, respiratory symptoms, and gastrointestinal complaints, and anaphylaxis in some cases (2-4).

All chemotherapy agents can cause HSRs^5^. Risk of inducing a lethal reaction can limit their therapeutic use in patients with severe reactions^6^. Regarding the increased frequency of HSR to drugs, and preference for first-line therapies in most of chemotherapeutic protocols, new treatment modalities for allergic patients have emerged.

Rapid drug desensitization (RDD) is available to patients with IgE-mediated HSRs^7^. This treatment modality protects patients against anaphylaxis; however, desensitization should be done at each drug administration to provide protection against anaphylaxis since the tolerance induced by desensitization is temporary (2,7,8).

Here we report 4 patients with anaphylaxis to chemotherapeutic agents, who have been successfully desensitized by using a standardized 12-step protocol (7).

*Case 1*

The first case was a 10-year old girl, who was receiving chemotherapy for the treatment of an Anablastic T cell lymphoma. She started LMB-96 protocol. During the course of chemotherapy, she developed urticaria, angioedema in her lips and tongue, dyspnea, cough, and dyspnea while receiving Etoposide (VP16) injection. The medication was discontinued immediately and the patient was diagnosed as anaphylaxis grade 3 (9). An immunology and allergy consult was done. The patient had no known allergy. Skin tests for Etoposide was performed according to drug provocation tests protocols (1), by a trained allergist and a trained fellow of allergy and clinical immunology, 4 weeks after the reaction. We prepared a dilution of 1mg/mL of Etoposide, then prepared three tenfold dilutions. Skin prick test (SPT) was performed with a 1:1000 dilution of Etoposide, histamine as a positive control and glycerin as a negative control. The SPT was negative. In serial intradermal tests (Id), we found a 9 mm wheal, 20 min after injection of 0.02 mL of 1:100 dilution compared to control. Considering the importance of Etoposide in this protocol, we decided to desensitize the patient. Therefore, the desensitization protocol was started according to a 12 step RDD protocol (7). The patient could tolerate all amount of Etoposide infusion during 6 hours. Of course the desensitization was repeated for the first day of Etoposide in the protocol. In this case the patient revealed urticaria, cough, and itching in the step 12, so we returned to the infusion rate administered in step 11 (40cc/h) for the remainder of the total dose (Table 1). In the following courses of her chemotherapy the same protocol was reapplied.

*Case 2*

The second case was an 8 year-old girl presented with right kidney tumor at the age of 5. After surgery, the diagnosis of Wilms tumor was confirmed. She underwent radiotherapy and chemotherapy. Then, the tumor was relapsed one year later with lung metastasis. The oncologist decided to start chemotherapy with radiotherapy and autologous hematopoietic stem cell transplantation. In relapse she received chemotherapy including Etoposide. During Etoposide infusion, the patient experienced flushing, angioedema, and hypotension (grade 2 anaphylaxis)^9^. She had no history of atopy and allergy. We evaluated the patient after 3 weeks. SPT was negative with the 1:1000 dilution of Etoposide, but in the Id tests, we found an 8 mm wheal, 20 min after injection of 0.02 mL of 1:1000 dilution compared to control. It was considered positive. Based on oncologist preference, the 12-step RDD protocol was planned for the patient. The patient needed to receive Etoposide for 5 consecutive days; therefore for the first day of Etoposide we applied the RDD protocol. In the 4 following days, she could tolerate the Etoposide without any reactions. Finally, she successfully tolerated all courses of Etoposide by RDD in the first day.

*Case 3*

The third case was a 12 year old boy presented with generalized lymphadenopathy since the age of 10. Excisional biopsy was performed from his cervical lymph nodes and showed a lymphoprolifrative disease. After hematology consult, the results of bone marrow aspiration and biopsy were in favor of acute lymphoblastic leukemia (T- cell ALL). Chemotherapy was started; he developed flushing, cough, and vomiting (anaphylaxis reaction grade 2) after receiving methotrexate (MTX) in the induction phase. The medication was discontinued immediately, and the anaphylaxis was treated. He had a history of a mild atopic dermatitis during infancy. We evaluated the patient 21 days after the reaction. We prepared a dilution of 1mg/mL of MTX then prepared three tenfold dilutions. Skin prick test (SPT) was performed with a 1:1000 dilution, histamine as a positive control and glycerin as a negative control, which was negative. In Id tests, we found an 8 mm wheal, 20 min after injection of 0.02 mL of the 1:100 dilution compared to control. The desensitization process was performed according to the 12 step RDD protocol. Finally the patient could receive all amounts of drug by this protocol.

*Case 4*

The 4^th^ case was an 11 year-old boy presented with generalized bone pain and pancytopenia. According to the results of bone marrow aspiration and biopsy, the diagnosis of ALL was established. Chemotherapy was started with VCR, daunorubicin, Prednisolone, L-asparaginase, and Intrathecal (IT) MTX and Cytarabine. He developed anaphylaxis (9) grade 1while receiving L-asparaginase in the last dose of induction. The medication was discontinued immediately and the anaphylaxis was treated. He had no known history of allergy. We prepared a dilution of 1unit/mL of L-asparaginase, then prepared three tenfold dilutions. SPT was performed with a 1:1000 dilution, histamine as a positive control and glycerin as a negative control, which was negative. In Id tests, we found a 10 mm wheal, 20 min after injection of 0.02 mLof the 1:100 dilution compared to control. An IgE-mediated drug allergy was confirmed by skin tests, and he became a candidate for L-asparaginase desensitization.We used the 12 step RDD protocol during consolidation period without any serious adverse reaction.

**Table 1:** Steps for protocol

**Step**	**Solution**	**Rate (cc/h)**	**Time (min)**
1	A	2	15
2	A	5	15
3	A	10	15
4	A	20	15
5	B	5	15
6	B	10	15
7	B	20	15
8	B	40	15
9	C	10	15
10	C	20	15
11	C	40	15
12	C	75	Till receiving the full dose

**Table 2:** The amount of target dose for each patient

	**Solution A**	**Solution B**	**Solution C**
Patient 1 (VP16)	2 mg*	20 mg	200 mg
Patient 2 (VP16)	1.6 mg	16 mg	160 mg
Patient 3 (MTX)	52 gr	520 mg	5200 mg
Patient 4 (L-asp)	900 u	9000 u	90000 u

* The amount of drug in 250 cc Dextrose Water 5%.

*Desensitization Procedure*

Drug desensitization should be performed with the drug that is necessary for therapy, in patient with anaphylaxis to the drug. Several desensitization protocols for HSRs to chemotherapy agents have been used (10-13). A 12-step protocol, generated by Castells ^7^, gradually increases the infusion rate and drug concentration to achieve the target dose over 5.8 h (Table 1).Three solutions; A, Band C, containing X/100 mg, X/10 mg, and X mg, respectively, were diluted in 250 mL of D5water and prepared according to the target dose for each patient (Table 2).Subsequently, the solutions were infused according to the protocol. Solution A was used for steps 1 to 4, B for steps 5 to 8, and C for steps 9 to12. The rate of the infusion was changed every 15 min. In the12^th^ and final step, constant rate of infusion was maintained to deliver the remainder of the total dose. Mild reactions including pruritus or pruritic rashes occurred in 2 patients, and they were treated by antihistamines. On the other hand, one of the patients experienced more symptoms in step 12, who could tolerate all her medication by slower rate of infusion.

## Discussion

Drug hypersensitivity reactions are adverse events resembling allergic reactions, which occur at therapeutic doses. Anticancer chemotherapeutics have the potential for acute HSR (5). Drug reactions might involve the immune system, through an IgE or non-IgE mediated mechanism^1^. HSR can be allergic or nonallergic, but the clinical manifestations are similar. RDD is effective when used appropriately, however it is often over utilized instead of performing a drug challenge. A skin test is used to assess the involvement of an IgE in the reaction and evaluate the treatment according to the algorithm by Castells (2,14).

RDD is both an acceptable approach and a high-risk treatment modality in whom, the offending agent is the first line chemotherapy. Desensitization induces a temporary tolerant state, which can only be maintained by continuous administration of the medication. Thus, for treatment like chemotherapy, the procedure must be repeated for every new course (7). 

The safety of this modality has been reported to be acceptable in some studies (2,7,15, 16). The reactions were often mild and the majority of them occurred during step 12, when patients were receiving the drug at the maximal rate and full concentration (7), like in patient number 1. The side effects were less frequent and less severe by repeating the protocol. Moreover, the pretreatment by antihistamines and/or antileukotriene has been useful in some cases (2,17).

Although the molecular basis of desensitization remains incompletely understood, mast cell models provided evidence of profound inhibitory mechanisms of cell activation during desensitization (7).

Basic research is needed to clarify the underlying mechanism of temporary tolerance, so that further interventions can improve the safety and efficacy of this approach.
